# The ColRS signal transduction system responds to the excess of external zinc, iron, manganese, and cadmium

**DOI:** 10.1186/1471-2180-14-162

**Published:** 2014-06-20

**Authors:** Kadi Ainsaar, Karl Mumm, Heili Ilves, Rita Hõrak

**Affiliations:** 1 Institute of Molecular and Cell Biology, University of Tartu, 51010 Tartu, Estonia

**Keywords:** ColRS two-component system, Metal tolerance, Histidine kinase, Signal perception, *Pseudomonas putida*

## Abstract

**Background:**

The ColRS two-component system has been shown to contribute to the membrane functionality and stress tolerance of *Pseudomonas putida* as well as to the virulence of *Pseudomonas aeruginosa* and plant pathogenic *Xanthomonas* species. However, the conditions activating the ColRS pathway and the signal(s) sensed by ColS have remained unknown. Here we aimed to analyze the role of the ColRS system in metal tolerance of *P. putida* and to test whether ColS can respond to metal excess.

**Results:**

We show that the ColRS system is necessary for *P. putida* to tolerate the excess of iron and zinc, and that it also contributes to manganese and cadmium tolerance. Excess of iron, zinc, manganese or cadmium activates ColRS signaling and as a result modifies the expression of ColR-regulated genes. Our data suggest that the genes in the ColR regulon are functionally redundant, as several loci have to be deleted to observe a significant decrease in metal tolerance. Site-directed mutagenesis of ColS revealed that excess of iron and, surprisingly, also zinc are sensed by a conserved ExxE motif in ColS’s periplasmic domain. While ColS is able to sense different metals, it still discriminates between the two oxidation states of iron, specifically responding to ferric and not ferrous iron. We propose a signal perception model involving a dimeric ColS, where each monomer donates one ExxE motif for metal binding.

**Conclusions:**

Several transition metals are essential for living organisms in certain amounts, but toxic in excess. We show that ColRS is a sensor system which detects and responds to the excess of physiologically important metals such as zinc, iron and manganese. Thus, the ColRS system is an important factor for metal homeostasis and tolerance in *P. putida.*

## Background

Metal ions are important catalytic and structural cofactors of proteins and are therefore necessary for the survival of all organisms. Among the metals found in enzymes, magnesium is the most abundant, followed by the transition metals zinc, iron and manganese. Other transition metals, such as cobalt, copper and nickel are less frequent in enzymes [1], but still important in a variety of cellular processes.

Although transition metals play a vital role in bacterial physiology, their excess can be toxic. For instance, iron can catalyze the formation of toxic reactive oxygen species via the Fenton reaction, which results in oxidative damage of proteins, lipids and DNA [2,3]. Highly competitive zinc and copper can easily outcompete other metals from metalloproteins [4] and therefore their free cytosolic concentrations are kept low [5,6]. To protect the cell from metal toxicity, bacteria most commonly use active metal efflux [7]–[9], but also metal chelation by specific proteins such as ferritin and metallothionein [10,11]. These processes, alongside with the repression of metal uptake systems, help maintain metal homeostasis in the condition of metal excess.

Given that maintenance of metal homeostasis is essential for bacteria, it is not surprising that they possess many regulatory pathways for sensing both the extra- and intracellular concentrations of metals. The cytosolic metal levels are monitored by different metalloregulators, such as Fur (for iron), Zur (for zinc), MntR (for manganese), etc., which control the expression of high-affinity metal uptake pathways that are able to supply the cell with the limiting metal [12]–[14]. Moreover, these systems also regulate the genes necessary for the detoxification of excess metals [15]. The external metal levels are detected primarily by transmembrane sensor proteins that belong to two-component signal transduction pathways. These sensors mediate the regulation of metal homeostasis via their cognate cytoplasmic response regulators. For instance, the PmrA-PmrB system in *Salmonella* monitors the amount of extracellular Fe^3+^ and Al^3+^ ions [16] and its activation leads to several lipopolysaccharide modifications [17], which alleviate metal toxicity by decreasing Fe^3+^ binding to the cell surface [18,19]. The PmrA-PmrB ortholog in *E. coli*, the BasS-BasR system, reacts to iron and zinc and regulates genes involved in membrane functions and stress response [20]. The ZraSR two-component system responds to high periplasmic Zn^2+^ and Pb^2+^ concentrations by up-regulating the expression of the periplasmic zinc-trapping chaperone ZraP, yet the role of this signal pathway in zinc resistance remains ambiguous [21,22]. These examples demonstrate that although some metal sensor systems can detect more than one metal, they are generally remarkably metal-specific, highlighting also the need for a large amount of sensor systems to maintain cellular metal homeostasis.

The genus *Pseudomonas* includes a great variety of widely distributed species that are known for their metabolic versatility and remarkable environmental adaptability [23]. Many pseudomonads are intrinsically highly resistant to different toxic compounds such as antibiotics, aromatics, detergents and heavy metals [24], which can be explained not only by their low outer membrane permeability and the presence of multiple efflux systems, but also by the large number of two-component signaling systems that are potentially able to shape the bacterial response to external stressors [25]. Interestingly, only a few metal resistance-regulating two-component systems have been characterized in pseudomonads so far. CzcRS has been described as a zinc-responsive system conferring resistance to zinc, cadmium and cobalt, but also to the antibiotic imipenem [26]. CopRS is a copper-activated signal system, which is required for copper resistance in *P. aeruginosa*[27], but also contributes to zinc resistance by activating the *czcRS* operon [28]. Contrarily, the CopRS ortholog of *P. fluorescens* seems to behave as a copper deficiency sensor that activates copper uptake when necessary [29]. This illustrates that even highly related sensor systems may sense and respond to different stimuli. Another example of that kind is PmrAB, which responds to external iron and alleviates iron toxicity in *Salmonella enterica*[16,18], but its ortholog in *P. aeruginosa* is not involved in iron resistance [30].

One of the well-conserved two-component systems in pseudomonads is the ColRS signaling pathway [31]. Its orthologs are also present in other environmental bacteria but seem to be absent from enteric bacteria. The ColRS system was first described as a root colonization factor of *P. fluorescens*[32]. Recent reports indicate that ColRS signaling is also important for the virulence of *P. aeruginosa*[33] and plant pathogenic *Xanthomonas* species [34,35]. ColRS deficiency results in pleiotropic effects in *P. putida,* including lowered phenol tolerance [36,37] and subpopulation lysis when bacteria grow under glucose limitation [38,39]. The phenotypic effects of ColRS deficiency as well as the identified target genes of the regulator ColR suggest that the ColRS system is involved in the regulation of membrane functionality [34,36,38,40,41]. However, so far the molecular basis of the membrane stress of the *colR* mutant as well as the signal sensed by ColS has remained unclear. Interestingly, recent reports suggest that the ColRS system may be involved in metal homeostasis, as it contributes to the copper tolerance of *X. citri*[34], cadmium tolerance of *X. campestris*[42] and multi-metal resistance of *P. putida* CD2 [43]. However, as the *P. putida* CD2 is a strain that is intrinsically highly resistant to different metal ions, the results cannot be easily extrapolated to other pseudomonads and the putative role of the ColRS system in metal resistance is yet to be determined.

Here we aimed to evaluate the impact of the ColRS system on metal tolerance of *P. putida* and to test whether metal excess could generate the activating signal for the sensor system. We demonstrate that ColRS signaling significantly contributes to *P. putida’s* zinc and iron tolerance, but is also slightly important in manganese and cadmium tolerance. All four of these metals can trigger ColS signaling, resulting in activation of the ColR regulon. We present evidence that a conserved ExxE motif in the periplasmic domain of ColS is required for sensing both zinc and iron, whereas only ferric and not ferrous iron can act as the signal for ColS.

## Results

### The ColRS system is required for growth in the excess of zinc, iron, manganese and cadmium

**Figure 1:**
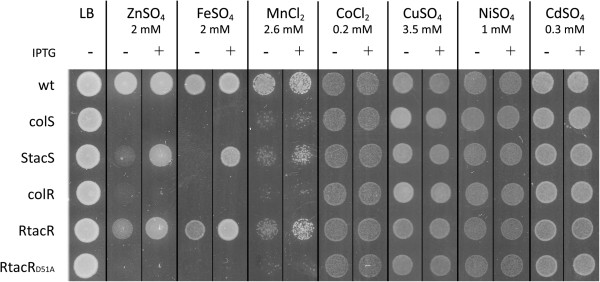
**Metal tolerances of different *P. putida *strains.***P. putida* wild-type strain PaW85 (wt), the *colS*-deficient strain (colS), *colS*-deficient strain complemented with the *colS* gene under the control of the inducible P_tac_ promoter (StacS), *colR*-deficient strain (colR), *colR*-deficient strain complemented with the *colR* gene under the control of the inducible P_tac_ promoter (RtacR) and *colR*-deficient strain complemented with the D51A mutant *colR* gene under the control of the inducible P_tac_ promoter (RtacR_D51A_) were grown on solid LB medium containing different metal salts for 20 hours at 30°C. ColS and ColR expression was induced with 0.5 mM IPTG indicated by “+”. Approximately 5000 cells were inoculated per spot.

To test whether the ColRS system is involved in metal resistance, we determined the MIC values of different transition metals for wild-type *P. putida* PaW85 and for its *colR-* and *colS-*deficient derivatives. In the liquid LB medium, the *colR* and *colS* mutants showed clearly increased sensitivity to zinc and iron compared to the parent strain (Table 1). The mutant strains were also slightly more sensitive to Mn^2+^ and Cd^2+^ but their resistance to Co^2+^, Cu^2+^ and Ni^2+^ resembled that of wild-type (Table 1). With the exception of Cd^2+^, similar results were observed when metal resistance was analyzed on LB solid medium – the growth of the *colR* and *colS* mutants was highly sensitive to the excess of zinc and iron, considerably impaired by manganese, but was not affected by other tested metals (Figure 1). Complementation of the *colS*- and *colR*-deficient strains with an extra copy of *colS* or *colR* genes under the control of the *tac* promoter and LacI repressor enabled normal growth of mutant bacteria under the condition of metal excess (Figure 1). The finding that the metal resistance of the RtacR strain was already restored without induction of *colR* expression with IPTG is in good correlation with previous results, as the *lacI*^*q*^*-P*_tac_*-colR* expression cassette has been shown to be highly leaky in *P. putida*[44]. In order to test whether the signal transduction between ColS and ColR is important for metal resistance, the *colR* mutant was complemented with ColR_D51A_, a phosphorylation-deficient variant of ColR [44]. As expression of ColR_D51A_ could not alleviate the metal sensitivity of the *colR* mutant (Figure 1), the signal transduction between ColS and ColR is clearly necessary for the growth of *P. putida* in high concentrations of zinc, iron and manganese.

**Table 1:** **MICs of different metals for *P. putida *** **parent strain PaW85 (wt) and its *colR *** **and *colS *** **knockouts**^**a**^

	**ZnSO**_**4**_	**FeSO**_**4**_	**CuSO**_**4**_	**CdSO**_**4**_	**CoCl**_**2**_	**MnCl**_**2**_	**NiSO**_**4**_
wt	5	5	6	1.5	1	8	3
colR	2	1.25	6	1	1	6	3
colS	2	1.25	6	1	1	6	3

^a^Numbers indicate millimolar concentrations.

### Genes of the ColR regulon respond to the excess of zinc in a ColS- and ColR-dependent manner

**Figure 2:**
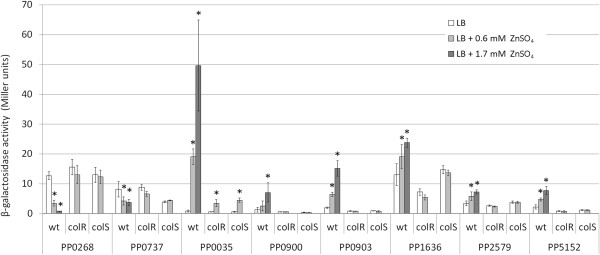
**ColR-regulated genes respond to excess of zinc.** β-galactosidase activities measured in *P. putida* wild-type (wt), *colR*- and *colS*-deficient strains (colR and colS, respectively) carrying the transcriptional fusions of PP0268, PP0737, PP0035, PP0900, PP0903, PP1636, PP2579 or PP5152 promoters with *lacZ* in the plasmid p9TT_B_lacZ. *P. putida* wild-type was grown in LB medium or LB where 0.6 mM or 1.7 mM ZnSO_4_ was added. *colR-* and *colS-*deficient strains were grown in LB or LB supplemented with 0.6 mM ZnSO_4_. Data (means with 95% confidence intervals) of at least three independent experiments are presented. Asterisks indicate statistically significant differences (p < 0.05, two-way ANOVA with post-hoc Tukey’s Unequal N HSD test) between values obtained in LB and in LB supplemented with ZnSO_4_.

Previous studies have identified several ColR-regulated genes in *P. putida*[36,40]. However, considering the quite modest effect of ColR in the regulation of those genes, it was proposed that the ColS-activating signal was not present under the conditions applied [40]. To test the hypothesis that metal excess could generate the activating signal for the ColS-ColR system, we investigated the expression of the ColR regulon genes under the conditions of high Zn^2+^. Analysis of known ColR-responsive promoters in wild-type *P. putida* revealed clear zinc-promoted induction of ColR-activated promoters (PP0035, PP0900, PP0903, PP1636) and inhibition of ColR-repressed ones (PP0268, PP0737) (Figure 2). Comparison of promoter activities of wild-type bacteria grown in the presence of either 0.6 or 1.7 mM ZnSO_4_ shows that zinc affects the ColR-regulated promoters in a concentration-dependent manner, resulting in a higher response at 1.7 mM ZnSO_4_ (Figure 2). The transcriptional effect of zinc clearly depended on the functionality of ColR and ColS because the zinc-responsiveness of promoters was not observed in *colR*- and *colS*-deficient strains (Figure 2). Only the PP0035 promoter displayed partial zinc-promoted but ColR-independent activation. Note that due to the high zinc-sensitivity of the *colR* and *colS* mutants, the promoter analysis in these strains was only possible in the presence of 0.6 mM but not 1.7 mM ZnSO_4_. In addition to promoters that were previously identified as ColR-regulated, we also studied whether some predicted members of the ColR regulon [40] could respond to zinc. Transcriptional analysis of several putative ColR target genes identified two new ColR-activated genes, PP2579 and PP5152, which responded to zinc in a ColR- and ColS-dependent manner (Figure 2). PP2579 and PP5152 code for two putative inner membrane proteins, the phosphoethanolamine transferase CptA and a conserved hypothetical protein, respectively, supporting the previously proposed role of the ColRS system in the regulation of membrane functionality. Together, our data indicate that ColRS signaling is activated by the excess of zinc, resulting in an altered expression of the ColR regulon genes.

### The excess of iron, manganese and cadmium can also affect the expression of the ColR regulon

**Figure 3:**
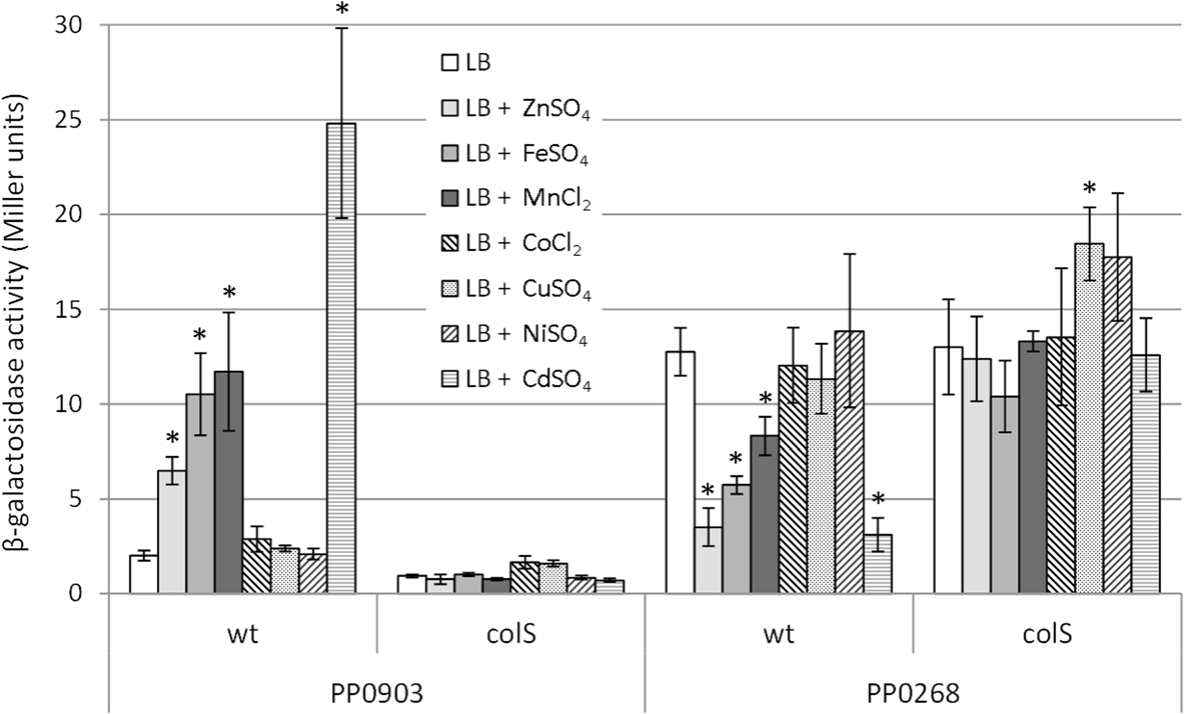
**ColR-regulated genes respond to excess of zinc, iron, manganese and cadmium.** β-galactosidase activities measured in *P. putida* wild-type (wt) and *colS*-deficient strain (colS) carrying the transcriptional fusions of PP0268 or PP0903 promoters with *lacZ* in the plasmid p9TT_B_lacZ. Bacteria were grown in LB medium and in LB containing either 0.6 mM ZnSO_4_, 0.15 mM FeSO_4_, 0.5 mM MnCl_2_, 0.1 mM CoCl_2_, 2 mM CuSO_4_, 0.5 mM NiSO_4_ or 0.2 mM CdSO_4_. Data (means with 95% confidence intervals) of at least three independent experiments are presented. Asterisks indicate statistically significant differences (p < 0.05, two-way ANOVA with post-hoc Tukey’s Unequal N HSD test) between values obtained in LB and in LB supplemented with metal salt.

Data presented above show that besides being important in zinc resistance, the ColRS system is also required for iron, manganese and cadmium resistance. To analyze whether other transition metals besides zinc can activate ColRS signaling, one ColR-activated (PP0903) and one ColR-repressed (PP0268) promoter was tested for metal responsiveness. The highest concentration of each metal tolerable to the *colS* mutant without growth retardation was used in this assay. Both ColR-regulated promoters respond to the excess of iron, manganese and cadmium, although the degree of response differs between different metals (Figure 3). To control whether iron-, manganese- and cadmium-promoted regulation of PP0903 and PP0268 indeed depends on ColRS activation, the promoters were also tested in the *colS*-deficient background. As the absence of ColS abolished the response of the promoters to metals (Figure 3), we conclude that four transition metals – zinc, iron, manganese and cadmium – can activate the ColRS signal transduction pathway. In accordance with MIC measurements, Co^2+^, Cu^2+^ and Ni^2+^ did not influence transcription from the ColR regulon genes, indicating that these metals do not produce the signal for the ColRS system.

### The expression of the *colRS* operon itself is not responsive to metal excess

**Figure 4:**
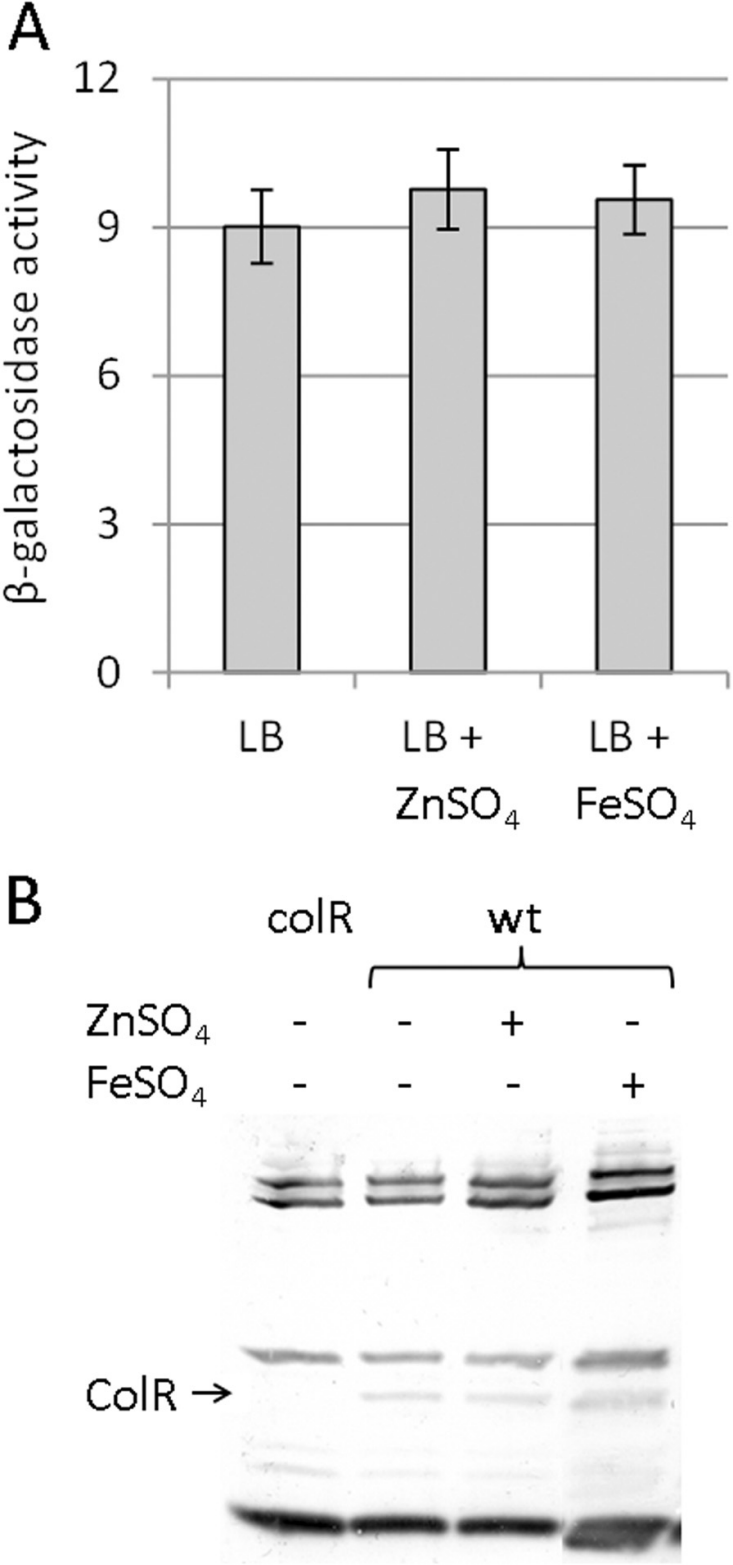
**Expression of ColR is not induced by metal stress. (A)** β-galactosidase activities measured in *P. putida* wild-type PaW85 strain carrying the transcriptional fusion of the *colRS* operon promoter with *lacZ* in the plasmid p9TT_B_lacZ. Bacteria were grown in LB medium and in LB containing 0.6 mM ZnSO_4_ or 0.15 mM FeSO_4_. Data (means with 95% confidence intervals) of at least four independent experiments are presented. **(B)** Western blot showing ColR expression in *P. putida* wild-type (wt) and *colR*-deficient strain (colR). Location of ColR is indicated with an arrow. Proteins were extracted from bacteria grown in LB medium and in LB containing 0.6 mM ZnSO_4_ or 0.15 mM FeSO_4_. All lanes contain 3 μg of total protein extract.

As genes of two-component systems involved in metal homeostasis are often induced in response to metal excess [26,45,46], we asked whether this could be the case with *colRS* as well. Previous studies have shown that despite being preceded by a ColR binding site, the *colR* promoter is not autoregulated and this site is associated only with the regulation of PP0900, located upstream of *colR*[40]. However, as this data was obtained under non-inducing conditions, we tested whether the expression of the *colRS* operon may respond to metal excess. Measurement of the β-galactosidase activity originated from the *colR-lacZ* transcriptional fusion showed that the *colR* promoter is influenced neither by 0.6 mM zinc nor by 0.15 mM iron (Figure 4A). Western blot analysis with anti-ColR antibodies confirmed that the abundance of ColR is not affected by the external excess of zinc or iron (Figure 4B).

### Impact of the ColR regulon genes on the zinc and iron resistance is highly redundant

As *colRS-*deficiency leads to sensitivity to several transition metals and these metals modulate the expression of the ColR regulon, we reasoned that the ColR-regulated genes should be important for metal resistance. To identify genes involved in metal resistance, we determined the MICs of metals for a set of knockouts of ColR regulon genes. We presumed that inactivation of the ColR-activated genes in wild-type background will decrease the metal resistance of bacteria and, vice versa, disruption of ColR-repressed genes will increase the metal resistance of the *colR*-deficient strain. Surprisingly, single gene or operon knockouts in the wild-type *P. putida* revealed no effect on iron (Table 2), manganese and cadmium (data not shown) resistance. The zinc resistance of these strains was also unaffected, except for a strain devoid of the PP0035-33 operon, which displayed a slightly lower MIC of zinc than the wild-type (Table 2). Furthermore, the disruption of ColR-repressed PP0268 and PP0737 in the *colR*-deficient strain did not influence the metal resistance of the *colR* mutant, either. In order to test whether the ColR regulon genes display functional redundancy, we constructed a set of strains devoid of several ColR-regulated genes and operons. Analysis of these mutants for metal susceptibility revealed that absence of at least four ColR-activated loci (PP0035-33, PP0900, PP0903-905, PP2579) was necessary to observe a significant decrease in the iron tolerance of *P. putida* (Table 2). As the iron tolerance of single, double and triple mutants was not changed, the reduced iron resistance of the quadruple mutant cannot be attributed to one particular locus and it rather indicates concert action of the ColR regulon genes. Analysis of zinc tolerance of strains devoid of multiple ColR-regulated genes showed that all strains lacking the PP0035-33 operon are slightly more sensitive to zinc, but no clear effect of other genes, with the exception of PP0900, could be recorded (Table 2). The detected MICs of all the strains for cadmium and manganese were similar to wild-type, indicating that none of the tested ColR regulon genes can significantly influence the tolerance of *P. putida* to these metals (data not shown). Importantly, even though some mutant strains displayed lower MIC values of iron and zinc compared to wild-type, none of them was as impaired as the *colR*-deficient strain. This can be explained by the weak effect of any single ColR-regulated locus on metal tolerance, but it may also indicate that the ColR regulon identified so far is yet incomplete.

**Table 2:** **MICs of zinc and iron for *P. putida *** **parent strain PaW85 (wt) and different knockout strains**

**Disrupted or deleted locus**	**(product, putative function)**	**ZnSO**_**4**_	**FeSO**_**4**_
		**mM**	**mM**
wt		5	5
colR		2	1.25
PP0035-PP0033	(LPS synthesis and modification)	4	5
PP0268	(porin OprE3)	5	5
PP0737	(PagL, LPS modification)	5	5
PP0900	(phospholipide metabolism)	5	5
PP0903-PP0905	(LPS modification)	5	5
PP1636	(DgkA, phospholipide metabolism)	5	5
PP2579	(CptA, LPS modification)	5	5
PP5152	(hypothetical protein)	5	5
PP0035-PP0033, PP0900		4	5
PP0035-PP0033, PP0903-PP0905		4	5
PP0035-PP0033, PP2579		4	5
PP0903-PP0905, PP2579		4	5
PP0035-PP0033, PP2579, PP0903-PP0905		4	5
PP0035-PP0033, PP2579, PP0903-PP0905, PP0900		3.5	3
PP0035-PP0033, PP2579, PP0903-PP0905, PP5152		4	5
colR, PP0268		2	1.25
colR, PP0737		2	1.25

### ColS possesses a putative iron binding motif in its periplasmic domain

**Figure 5:**
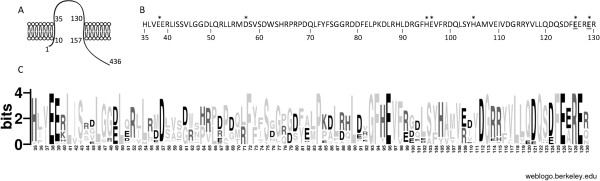
**Sequence analysis of the periplasmic domain of ColS. (A)** Localization of the ColS protein in the inner membrane. Numbers correspond to the amino acid residues in ColS sequence showing the first and the last amino acid of ColS, its transmembrane domains and the periplasmic domain. **(B)** Amino acid sequence of the periplasmic domain of *P. putida* ColS. Glutamic acids of the putative iron binding motif are underlined. Asterisks indicate the amino acid residues mutated in this study. **(C)** Conservation of ColS’s periplasmic domain. Sequence logo for ColS periplasmic domain was created with the WebLogo server using 47 ColS sequences annotated in the Pseudomonas Genome Database. The acidic and basic amino acids are indicated in black and dark grey, respectively. Other amino acids are presented in light grey. The degree of sequence conservation at each position is indicated as the total height of a stack of letters, measured in arbitrary “bit” units, with a theoretical maximum of 4.3 bits at each position.

ColS is a canonical membrane kinase with two transmembrane domains connected by a 96 amino acid periplasmic loop, which is most probably involved in signal recognition (Figure 5A). Metal-sensing sites of proteins are composed of several metal-binding residues, which are most often glutamic acid, aspartic acid and histidine [47]. To predict the periplasmic amino acids that are putatively involved in metal sensing by ColS, we aligned the periplasmic regions of 47 annotated ColS orthologs represented in the Pseudomonas database [31]. From 96 putative periplasmic residues, 14 turned out to be conserved among all analyzed ColS proteins and four of these identical residues were glutamic acids in positions 38, 96, 126 and 129 (Figure 5 B and C). Notably, E126 and E129 appeared in the most conserved F**E**xR**E** sequence, which resembles the EXXE motif implicated in direct binding of iron in several proteins [16,48,49]. Thus, we hypothesized that this motif may bind iron in ColS. Considering that the ColRS system also responds to zinc and that histidine is a particularly important residue in coordination of Zn^2+^ in several zinc-binding proteins [12], we also analyzed the conservation of five periplasmic His residues found in ColS of *P. putida*. The most conserved histidine, H35, was present in 44 out of 47 ColS proteins (Figure 5B). If the eight less conserved ColS orthologs were omitted from the alignment, then also H95 and H105 appeared to be conserved.

### Conserved glutamic acids of the ExxE motif in ColS are necessary for metal-promoted activation of a ColR-regulated promoter

To examine the role of the conserved glutamic acids and histidines in the signaling ability of ColS, the ColS variants possessing a substitution mutation (H35A, E38Q, H95A, E96Q, H105A, E126Q or E129Q) in the periplasmic domain were cloned under the control of the *tac* promoter. We also constructed a ColS derivative carrying the replacement of aspartic acid at position 57 (D57N) as well as ColS with both E126Q and E129Q replacements. The expression cassettes for the mutant ColS variants were introduced into the chromosome of the *colS-*deficient strain and the abundance of the overexpressed ColS proteins was analyzed with anti-ColS antibodies. However, due to the low sensitivity of antibodies we could detect neither the wild-type nor the overexpressed level of ColS (data not shown). Thus, the abundance of ColS in *P. putida* seems to be low, even when expressed from the IPTG-inducible *tac* promoter.

**Figure 6:**
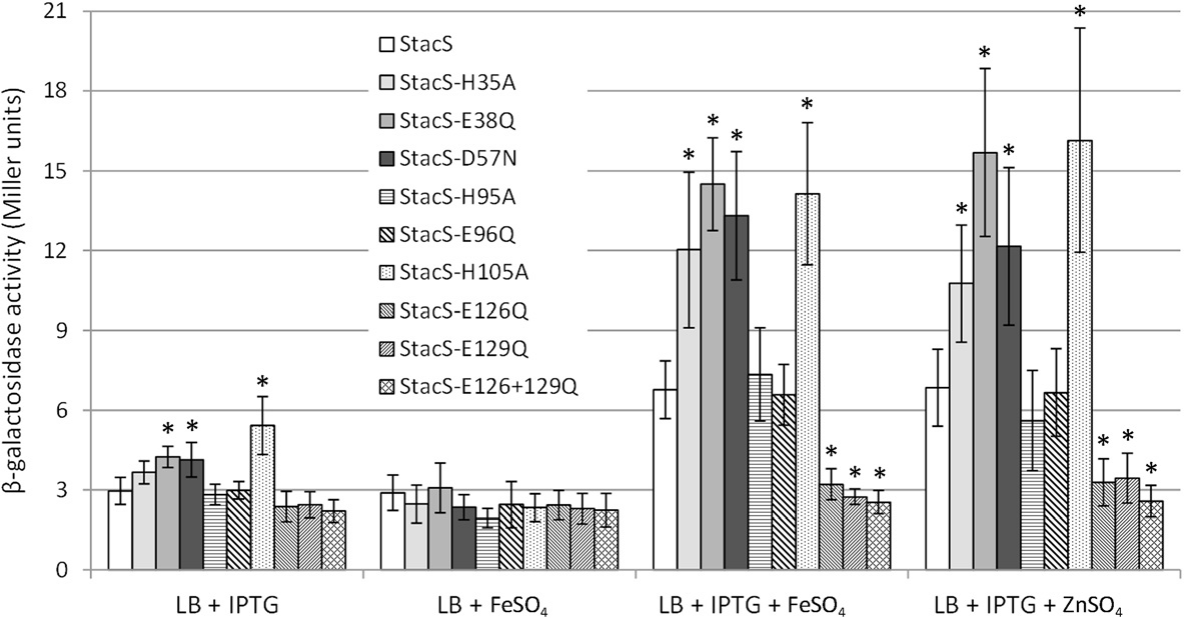
**Conserved glutamic acids of the ExxE motif in ColS are necessary for metal-promoted activation of a ColR-regulated promoter.** β-galactosidase activities measured in *P. putida colS*-deficient strain complemented with either the wild-type *colS* (StacS) or the *colS* variants carrying single substitutions of H35A, E38Q, D57N, H95A, E96Q, H105A, E126Q, E129Q or the double substitutions of E126Q and E129Q under the control of the inducible P_tac_ promoter. All strains carry the transcriptional fusion of the PP0903 promoter with *lacZ* in the plasmid p9TT_B_lacZ. Bacteria were grown in LB medium containing 0.1 mM IPTG or 0.15 mM FeSO_4_ or 0.1 mM IPTG and 0.15 mM FeSO_4_ or 0.1 mM IPTG and 0.6 mM ZnSO_4_. Data (means with 95% confidence intervals) of at least six independent experiments are presented. Asterisks indicate a statistically significant difference (p < 0.01, Student’s *t*-test) between the StacS strain and a strain carrying a mutant ColS in a particular medium.

Analysis of metal-promoted activation of ColR-regulated PP0903 revealed that responsiveness of ColS to both iron and zinc was lost when either of two conserved glutamates in the F**E**ER**E** motif were mutated (Figure 6). Bacteria expressing single mutants ColS_E126Q_ and ColS_E129Q_ as well as the double mutant ColS_E126Q/E129Q_ displayed a basal expression level of PP0903 promoter, which was comparable to the non-induced controls when either IPTG or metal was absent from medium (Figure 6, data for LB + 0.6 mM ZnSO_4_ are not shown). None of the other amino acid substitutions could decrease the signaling ability of ColS. Quite the contrary, some ColS mutants (ColS_H35A_, ColS_E38Q_, ColS_D57N_, and ColS_H105A_) demonstrated an even higher responsiveness to both zinc and iron than wild-type ColS. Interestingly, analysis of ColS_E38Q_, ColS_D57N_, and ColS_H105A_ mutants in the medium which was supplemented with IPTG but not with metals (Figure 6) revealed partial activation of the PP0903 promoter. These data indicate that the F**E**ER**E** motif is implicated in signal perception, but also suggest that amino acids H35, E38, D57 and H105 regulate the metal-sensing ability of ColS. The alternative explanation for the signal-blind phenotype of some of the mutant ColS proteins could be their lower stability. However, we do not believe that a single amino acid substitution in the periplasmic domain of a membrane protein can essentially affect its stability as there are several indications that membrane proteins are remarkably tolerant to substitution mutagenesis [50,51].

### ColS specifically responds to ferric iron

**Figure 7:**
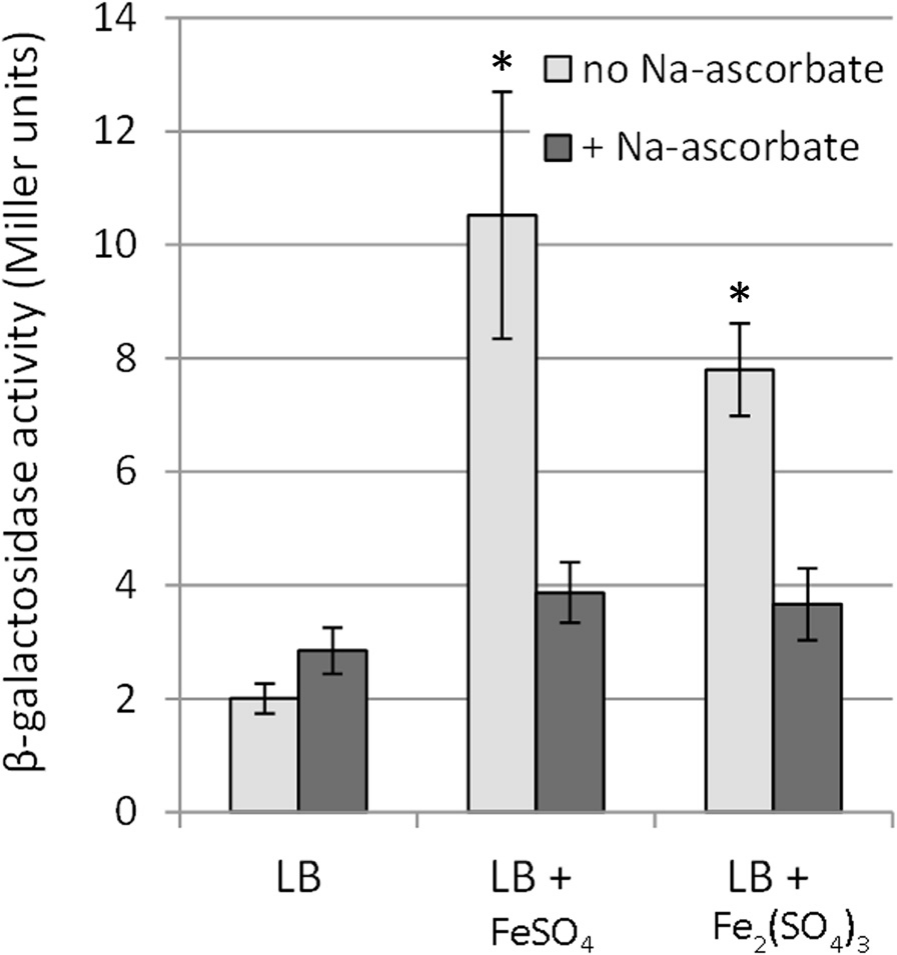
**ColS responds to ferric iron.** β-galactosidase activities measured in *P. putida* wild-type PaW85 strain carrying the transcriptional fusion of the PP0903 promoter with *lacZ* in the plasmid p9TT_B_lacZ. Bacteria were grown in LB medium and in LB containing 0.15 mM FeSO_4_ or 0.075 mM Fe_2_(SO_4_)_3_ with and without 0.5 mM Na-ascorbate. Data (means with 95% confidence intervals) of at least six independent experiments are presented. Asterisks indicate a statistically significant difference (p < 0.05, two-way ANOVA with post-hoc Bonferroni’s multiple comparison test) between values obtained in media containing no Na-ascorbate and media supplemented with Na-ascorbate.

To our knowledge, there are three bacterial two-component systems, PmrA/PmrB, FirR/FirS, and BqsR/BqsS, which can sense extracellular iron [16,46,52]. All of these signaling systems can discriminate between ferrous (Fe^2+^) and ferric (Fe^3+^) ions. While PmrB of *Salmonella enterica* specifically responds to Fe^3+^[16], BqsS of *Pseudomonas aeruginosa* and FirS of *Haemophilus influenzae* are activated by Fe^2+^ only [46,52]. In all the experiments presented above we used ferrous sulphate (FeSO_4_) as the source of iron, however, the ferrous ions are easily oxidized to ferric ions in the solutions. In order to discriminate whether Fe^2+^ or Fe^3+^ can activate ColS, the metal-promoted activation of the PP0903 promoter was tested in media supplemented either with ferrous or ferric sulphate. To keep iron in a reduced state we also performed experiments in the presence of 5 mM sodium ascorbate. Data in Figure 7 show that transcription from the PP0903 promoter can be induced both by ferrous and ferric sulphate. However, considering that sodium ascorbate can suppress the responses elicited by either metal salt, we deduce that ferric iron is the signal sensed by ColS. This conclusion was further supported by the finding that the same amount of sodium ascorbate could not affect the zinc-promoted activation of ColS (data not shown).

## Discussion

The controversial nature of biologically important transition metals requires constant monitoring of their concentrations to avoid potential toxic effects of metals. In this study, we demonstrate that the ColRS two-component system acts as a sentinel for external levels of zinc, iron, manganese, and cadmium. Metal-promoted signaling of ColRS system results in the activation of the ColR regulon, which contributes to metal tolerance of *P. putida*.

The finding that the ColRS system is involved in metal tolerance is consistent with previous reports as the ColRS system has been shown to promote heavy metal tolerance of *P. putida* CD2 [43], cadmium tolerance of *Xanthomonas campestris*[42], and copper tolerance of *X. citri*[34]. Comparison of our metal tolerance data for *P. putida* PaW85 with those previously published for *P. putida* CD2 [43] revealed that the absence of the ColRS system results in different outcomes in these two strains. While the disruption of ColRS signaling in *P. putida* PaW85 increases the sensitivity of bacteria only to the excess of zinc, iron, manganese and cadmium, the ColRS-deficient *P. putida* CD2 also displays higher susceptibility to copper, cobalt and nickel. However, one should consider that *P. putida* CD2 was isolated from sewage sludge as a cadmium-resistant bacterium [43] and this strain is substantially more tolerant to metals than *P. putida* PaW85. Therefore, it is not surprising that these two *P. putida* strains behave somewhat differently from each other although their *colRS* operons are almost identical. The ColRS systems of *X. campestris* and *X. citri* are distantly related orthologs of the ColRS of *P. putida*, as judged by the 57% identity of ColR and only about 26-27% identity of ColS proteins. Furthermore, while the C-terminal kinase domains of the ColS proteins of *P. putida* and *Xanthomonas* strains are considerably similar, the N-terminal sensing domains are remarkably divergent (not shown). This suggests that the signal recognition mechanism of ColS in *Xanthomonas* may be different from that in *P. putida*.

The ColR regulon genes responded to the physiologically important zinc, iron and manganese, but also to the dispensable and highly toxic cadmium. The ColRS-dependent response to the excess of zinc and iron is obviously highly relevant because disruption of the ColRS system remarkably decreased both the iron and zinc tolerance of *P. putida* (Table 1). We also showed that the functionality of the ColR regulon is important in iron and zinc tolerance, although the impact of any single gene alone is weak and the regulon genes appear to act redundantly (Table 2). Differently from zinc and iron, the MICs of manganese and cadmium for the ColRS-deficient strain were only slightly lower than that of the wild-type, suggesting that the activation of the ColR regulon by these metals is not as important for *P. putida* as the response induced by zinc or iron. However, manganese is considered less harmful than zinc or iron as it is less able to replace other metals in their complexes and it does not produce hydroxyl radicals like iron [4,53]. This and other possible ColRS-independent manganese tolerance mechanisms could be the reasons why inactivation of ColRS signaling does not result in major effects in the manganese tolerance of *P. putida*. Intriguingly, cadmium promoted the strongest activation of the ColR regulon genes but, despite that, the cadmium tolerance of *colRS* mutants was hardly affected, being observable only in liquid and not in solid medium (Figure 1, Table 1). This suggests that the ColRS system is of little importance under cadmium stress and other resistance mechanisms exist that confer the cadmium tolerance of *P. putida*. The most probable candidates could be the several cadmium-induced efflux systems which are known to contribute to cadmium resistance of *P. putida*[54]. Given all these data, we suggest that although manganese and cadmium can activate the ColRS signaling, the primary role of ColRS is to maintain zinc and iron homeostasis.

The metal-controlled ColR regulon includes genes and operons putatively involved in the synthesis and/or modification of LPS or in the metabolism of phospholipides (Figure 2, Table 2). Notably, deletion of most of the ColR regulon genes individually did not change the metal sensitivity of bacteria and inactivation of at least four loci was necessary to observe their effect on metal tolerance. The only locus that could significantly contribute to zinc, but not iron tolerance, is the PP0035-PP0033 operon that codes for three membrane proteins. This operon is most probably involved in LPS modification because PP0033 and PP0034 code for proteins homologous to ArnT (referred also as PmrK or PbgE) and ArnC, respectively, which decorate lipid A with the cationic sugar 4-amino-4-deoxy-L-arabinose [55,56]. The addition of 4-amino-4-deoxy-L-arabinose to lipid A decreases the negative charge of LPS, which has been demonstrated to increase the resistance of *Salmonella* to cationic antimicrobial peptides and also to Fe^3+^ and Al^3+^[17,18]. Analogously, we consider that the impact of PP0033 and PP0034 in metal tolerance may rely on their ability to modify LPS. Notably, there is another gene in the ColR regulon, which can putatively decrease the negative charge of cell surface by LPS modification. The ColR-activated PP2579 encodes a protein homologous to CptA phosphotransferase, which catalyzes the phosphoethanolamine addition to the LPS core [57]. Interestingly, genes responsible for the addition of 4-amino-4-deoxy-L-arabinose and phosphoethanolamine to LPS in *Salmonella* are regulated by the PmrAB two-component system [57]–[59], which, like ColRS, responds to external iron [16]. This suggests that the mechanism how ColRS system impacts the metal tolerance of *P. putida* partly resembles that of PmrAB, where modification of LPS plays a major role in protecting bacteria from metal toxicity [18,60,61]. However, we want to emphasize that the effect of PP0035-PP0033 and PP2579 in metal tolerance is rather low and that the ColR-controlled metal tolerance is actually provided by the joint action of the whole regulon.

Several signaling systems which regulate bacterial response to external metals are induced by the same environmental cue they respond to. For example, expression of *pmrAB* in *Salmonella* is induced by iron, *basSR* in *E. coli* is induced by iron and zinc, *bqsRS* and *czcRS* in *P. aeruginosa* are upregulated by iron and cadmium, respectively [16,26,45,46]. Differently from these systems, the expression of *colRS* is not affected by metals and the ColRS-promoted response to metal excess only involves activation of the signal transduction between the system counterparts and the resulting changes in the expression of the ColR regulon genes. This suggests that the basal constitutive expression level of the *colRS* operon is sufficient to guarantee an appropriate response to metal stress.

Mutational analysis of ColS indicates that a conserved ExxE motif of the periplasmic loop of the sensor kinase is required for sensing both iron and zinc, because substitution of either of the conserved glutamic acid residues in this motif abolishes the ability of ColS to respond to both metals and to promote the activation of the ColR regulon (Figure 6). The ExxE motif has been demonstrated to bind iron in several eukaryotic and prokaryotic proteins, including, for instance, the iron transporter FTR1 in *Saccharomyces cerevisiae*[48], the iron sensor PmrA in *Salmonella enterica*[16], the iron- and heme-binding HbpS in *Streptomyces reticuli*[49]. Interestingly, as far as we know, there are no previous reports demonstrating that the iron-binding ExxE motif could also bind zinc. However, given that glutamates have been found to coordinate the zinc ion in many zinc-binding proteins [62], the zinc-binding ability of the ExxE motif is actually anticipated and one may suspect that other ExxE motif-containing proteins could bind zinc as well. Mutational analysis of ColS also showed that while the ExxE motif is necessary for iron and zinc sensing, the other conserved amino acids in the ColS periplasmic domain are important for the regulation of the signaling ability of ColS. Besides, it is remarkable that none of the amino acid substitutions outside the ExxE motif decreased the signaling ability of ColS and some even increased it. For example, the substitutions H35A, E38Q, D57N and H105A significantly increased the responsiveness of ColS to both iron and zinc (Figure 6), suggesting that these positions are important for keeping ColS in the inactive state and for preventing premature signaling under non-induced conditions. Notably, the mutations E38Q, D57N and H105A resulted in somewhat higher signaling of ColS even without metal stress, implying that the conformations of the ColS_E38Q_, ColS_D57N_ and ColS_H105A_ are changed, allowing the higher basal kinase activity of the proteins. Interestingly, another clue suggests that the ColS region containing H105 is important for regulation of ColS activity by keeping the sensor in the inactive form. Recently, the ColRS system was shown to support the polymyxin resistance of *P. aeruginosa*, whereas the mutant ColS possessing a substitution A106V seemed to enhance the polymyxin resistance of a *P. aeruginosa* clinical isolate [63]. It is tempting to speculate that the ColS_A106V_ in *P. aeruginosa*, analogously to our ColS_H105A_, may also be more active than wild-type ColS, resulting in higher activation of the ColR regulon and, as a consequence, higher polymyxin resistance of *P. aeruginosa*.

**Figure 8:**
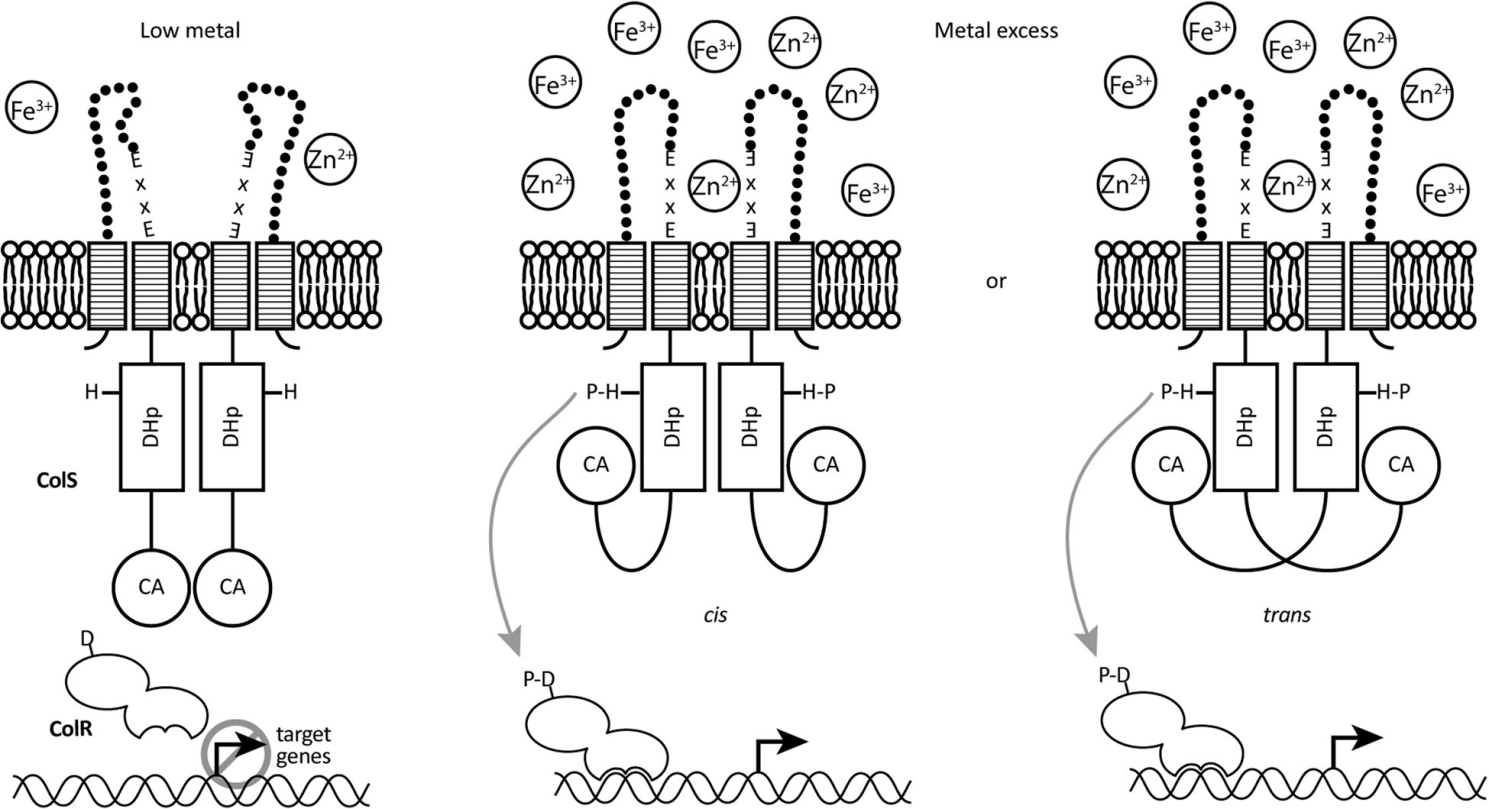
**Model of signal recognition and activation of the ColRS system.** When Zn^2+^ or Fe^3+^ concentration is low, metal ions are not bound by the periplasmic domain of ColS and ColR is not phosphorylated. When *P. putida* experiences metal excess, a Zn^2+^ or Fe^3+^ ion binds with four glutamic acids of two ExxE motifs from two ColS proteins. Ion binding changes ColS conformation and the conserved histidine (H) in the dimerization and histidine phosphotransfer domain (DHp) is autophosporylated by the catalytic domain (CA) of ColS. Both *in cis* and *in trans* phosphorylation mechanisms are presented. Phosphate group is subsequently transferred from ColS to ColR and as a result ColR becomes active as a transcription regulator.

It has been shown that four glutamic acids of two ExxE motifs located in different monomers participate in coordinating of iron in the octameric HbpS [49]. Given that the zinc ion also has a marked preference for tetrahedral coordination geometry [62], two ExxE motifs should be involved in binding of zinc as well. As ColS possesses only one conserved ExxE motif in its periplasmic domain, we propose a model involving dimeric ColS, where, analogous to HbpS, each monomer donates one ExxE motif for metal binding (Figure 8). The ExxE motif of ColS is located in the most C-terminal part of the periplasmic domain, positioned close to the second transmembrane domain. Therefore, it is most probable that the two ExxE motifs are located closely in the ColS dimer and are oriented towards each other in the interface of adjacent subunits (Figure 8). If the extracellular concentration of Fe^3+^ or Zn^2+^ exceeds a certain threshold level, the ColS dimer will bind the metal ion, resulting most probably in a conformational change and autophosphorylation of ColS. Subsequent signal transduction to ColR and activation of the ColR regulon leads to modifications in the cell membrane, which helps bacteria cope with metal excess. Considering that the metal-sensing ExxE motif of ColS is highly conserved in all sequenced pseudomonads, it suggests that the other ColRS systems may have a similar metal-sensing mechanism as well.

## Conclusion

The most important result of the current study is that for the first time, the signal for a ColRS two-component system has been determined. We show that ColS is a metal sensor which is activated when the growth medium contains excess iron, zinc, manganese or cadmium. Our data indicate that a conserved ExxE motif in the periplasmic domain of ColS is involved in both zinc and iron sensing and is able to distinguish between different iron ions, responding only to ferric iron. The finding that the ExxE motif is involved in zinc sensing is novel as it has previously been reported to bind iron only [16,48,49]. We show that the metal-promoted activation of ColS results in the activation of the ColR regulon which is necessary to protect the bacteria from metal-mediated toxicity. This adaptive system could be highly beneficial for soil bacteria, such as *P. putida* and other pseudomonads, as well as *Xanthomonas* species, as they may experience elevated metal concentrations in their native environments.

## Methods

### Bacterial strains, plasmids, and media

The bacterial strains and plasmids used are listed in Additional file 1. All *P. putida* strains are derivatives of PaW85 [64], which is isogenic to the fully sequenced KT2440 [65]. Bacteria were grown in lysogeny broth (LB). To generate metal stress, the LB medium was supplemented with the following metal salts: ZnSO_4_, FeSO_4_, Fe_2_(SO_4_)_3_, CuSO_4_, NiSO_4_, CdSO_4_, MnCl_2_, and CoCl_2_. When selection was necessary, the growth medium was supplemented with ampicillin (100 μg ml^-1^), kanamycin (50 μg ml^-1^) or streptomycin (20 μg ml^-1^) for *E. coli* and benzylpenicillin (800 μg ml^-1^), kanamycin (50 μg ml^-1^) or streptomycin (100 μg ml^-1^) for *P. putida. E. coli* was incubated at 37°C and *P. putida* at 30°C. Bacteria were electrotransformed according to the protocol of Sharma and Schimke [66].

### Construction of plasmids and strains

Oligonucleotides used in PCR amplifications are listed in Additional file 2. For complementation of the *colS*-deficient strain with an IPTG-inducible copy of *colS*, the *colS* gene was first amplified from the *P. putida* PaW85 chromosome with primers ColSSal and ColSHincII. The PCR fragment was cut with SalI and HincII and cloned into SalI-SmaI-opened pBRlacItac. The *lacI*^*q*^*-P*_*tac*_*-colS* cassette was excised from the plasmid pBRlacItac/colS with BamHI and Acc65I, and ligated into the corresponding sites of the plasmid pUC18Not to obtain pUCNot/lacItaccolS. Finally, the *colS* expression cassette was subcloned as a NotI fragment into the miniTn7 delivery plasmid pBK-miniTn7-ΩSm. For the construction ColS_H35A_, ColS_E38Q_, ColS_D57N_, ColS_H95A_, ColS_E96Q_, ColS_H105A_, ColS_E126Q_, ColS_E129Q_ and ColS_E126Q/E129Q_ expression cassettes, the site-directed mutagenesis of wild-type *colS* was performed using two sequential PCRs and the plasmid pUCNot/lacItaccolS as a template. In the first PCR, one primer carried the substitution mutation and the other was either Smut1 or Smut2 (see Additional file 3). The product of the first PCR served as a reverse primer for Smut1 or Smut2 in the second PCR. The product of the second PCR was treated with DpnI, Mva1269I and Bpu1102I, and ligated into the Mva1269I-Bpu1102I-opened pUCNot/lacItaccolS. After verification of designed mutations by sequencing, the expression cassettes with the mutated *colS* gene were subcloned into the NotI site in plasmid pBK-miniTn7-ΩSm. The pBK-miniTn7-ΩSm derivatives, bearing either wild-type or mutant *colS* expression cassette, were introduced into *P. putida colS*-deficient strain by co-electroporation together with the helper plasmid pUXBF13. Presence of the expression cassette in the *attTn7* site of the *colS-*deficient strain was verified by PCR.

For construction of *P. putida* derivatives devoid of PP0268, PP0900, PP1636 or PP5152, the loci were disrupted with the streptomycin resistance gene. PP0268, PP0900, PP1636 or PP5152 were amplified with primer pairs oprE3Bam + oprE3Xho, 900Kpn + colRATGXho, PP1635lopp + PP1636Kpn and 5152lopp + 5153lopp, respectively. PP0268-containing PCR fragment was treated with BamHI (blunt-ended with Klenow DNA polymerase) and XhoI and cloned into pBluescript KS. The central 700-bp region of PP0268 in pKS/268 was excised with HincII and Eco47III and replaced with the Sm^r^ gene cut from pUTmini-Tn5Sm/Sp with VspI. The obtained 268::Sm sequence was subcloned as an EcoRI-Acc65I fragment into pGP704L. PP0900-containing PCR fragment was treated with Eco147I and Acc65I and cloned into the SmaI-Acc65I-opened pBluescript KS. Next, the central 87-bp EheI-Eco130I sequence in pKS/900 was replaced with the Sm^r^ gene and the resulting 900::Sm sequence was subcloned into pGP704L using SacI and Acc65I. The PP1636-containing PCR fragment was cloned into pBluescript KS as a HindIII-Acc65I fragment. The central 143-bp Mva1269I-ClaI region of PP1636 in pKS/1636 was replaced with the Sm^r^ gene and the 1636::Sm sequence was inserted into pGP704L using SacI and Acc65I. The PP5152-containing PCR fragment was cloned into pBluescript KS as a PstI-Acc65I fragment. The central 377-bp HincII-Bpu1102I fragment of PP5152 in pKS/5152 was replaced with the Sm^r^ gene and the 5152::Sm sequence was inserted into pGP704L using XbaI and PvuII. The interrupted PP0268, PP0900, PP1636 or PP5152 genes were inserted into the chromosome of *P. putida* PaW85 and its knockout derivatives by homologous recombination. Plasmids p704L/268::Sm, p704L/900::Sm, p704L/1636::Sm or p704L/5152::Sm were conjugatively transferred from *E. coli* CC118 λ*pir* into *P. putida* using the helper plasmid pRK2013. The gene knockout strains were verified by PCR analysis.

For generation of deletion strains devoid of single or multiple genes, the pEMG-based plasmids were constructed according to the protocol described elsewhere [67]. The upstream and downstream regions (about 500 bp) of the gene(s) to be deleted were amplified separately and then joined into one fragment by overlap extension PCR. For construction of the plasmids pEMG-Δ35-33, pEMG-Δ737, pEMG-Δ903-905 and pEMG-Δ2579, the PCR fragments of about 1 kb were cut with SalI and EcoRI, BamHI and EcoRI, Acc65I and SacI, and SalI and SacI, respectively, and ligated into the corresponding sites of the plasmid pEMG. The obtained pEMG plasmids were delivered to *P. putida* PaW85 or its knockout derivative strains by electroporation and after 3 hours of growth in LB medium the bacteria were plated onto LB agar supplemented with kanamycin. Kanamycin-resistant co-integrates were selected and electrotransformed with the I-SceI expression plasmid pSW(I-SceI). In order to resolve the cointegrate, the plasmid-encoded I-SceI was induced with 1.5 mM 3-methylbenzoate overnight. Kanamycin-sensitive colonies were selected and the deletions of PP0035-PP0033, PP0737, PP0903-PP0905 or PP2579 were verified by PCR. The plasmid pSW(I-SceI) was eliminated from the deletion strains by growing them overnight in LB medium without antibiotics.

To construct the transcriptional fusions of the PP2579 and PP5152 promoters with *lacZ*, the upstream regions of PP2579 and PP5152 were amplified from the *P. putida* PaW85 chromosome with primers PP2579alg and PP2580alg, and 5152alg and 5153lopp, respectively. The resulting PCR fragments were treated with HindIII and inserted into HindIII-opened p9TT_B_lacZ.

### Metal tolerance plate assay

Metal tolerance was evaluated on LB agar plates containing different concentrations of metal salts (concentrations are specified in Results). The LB-grown overnight cultures were tenfold serially diluted, spotted onto plates as 5 μl drops and incubated at 30°C for 20 hours.

### Determination of minimal inhibitory concentrations (MICs) of metals

The MICs of different metals were determined for bacteria growing in microtiter plates. The microtiter plate wells containing serial dilutions of metal salts in 100 μl LB medium were inoculated with about 1 × 10^6^ cells of bacterial culture pre-grown overnight in LB medium. Microtiter plates were incubated with shaking at 30°C for 24 h, after which the OD_580_ was measured. The MIC was defined as the lowest concentration of metal that allowed no bacterial growth. For each metal and bacterial strain, at least three independent experiments were carried out.

### β-galactosidase assay

Enzyme activities were measured from bacteria grown overnight in LB or in LB supplemented with different metal salts (concentrations are specified in Results). β-galactosidase activity was assayed according to a previously described protocol [68].

### Western blotting

Cell lysates were prepared from bacteria grown overnight in LB or in LB supplemented with either 0.6 mM ZnSO_4_ or 0.15 mM FeSO_4_. Equal amounts of total protein (3 μg) were separated by Tricine-SDS-PA gel electrophoresis, followed by protein transfer to a nitrocellulose membrane. For Western blotting, the membranes were probed with ColR-specific polyclonal antibodies, followed by treatment with alkaline phosphatase-conjugated goat anti-rabbit immunoglobulin G. The blots were developed using bromochloroindolyl phosphate/nitro blue tetrazolium (BCIP/NBT).

## Competing interests

The authors declare that they have no competing interests.

## Author’s contributions

KA carried out all enzyme activity measurements, performed ColS mutagenesis and tolerance plate assays. KM performed MIC measurements. KA, RH and HI constructed the plasmids and strains. RH conceived, designed and coordinated experimental work and manuscript editing. All authors read and approved the final manuscript.

## Supplementary Material

Additional file 1: Table S1 Bacterial strains and plasmids. Click here for file

Additional file 2: Table S2 The oligonucleotides. Click here for file

Additional file 3: Table S3 The oligonucleotide pairs used in two sequential PCRs for site-directed mutagenesis of *colS.* Click here for file
