# Supplementary material for: The ColRS signal transduction system responds to the excess of external zinc, iron, manganese, and cadmium
Source: BMC Microbiol. 2014 Jun 20;14:162. doi: 10.1186/1471-2180-14-162 (PMC4074579; doi:10.1186/1471-2180-14-162)
Supplement: Additional file 1: Table S1 — Bacterial strains and plasmids. [file 1471-2180-14-162-S1.docx]

Additional Table S1. Bacterial strains and plasmids

| Strain or plasmid | Genotype or characteristics | Source or reference |
| --- | --- | --- |
| *Escherichia* *coli* |  |  |
| DH5α | *supE44* Δ*lacU169* (α80 *lacZ*ΔM15) *recA1 endA1 hsdR17 thi-1 gyrA96 relA1* | [[1](#_ENREF_1)] |
| CC118λpir | Δ(*ara-leu*) *araD* Δ*lacX74 galE galK phoA20 thi-1 rpsE rpoB argE*(Am) *recA1* λ*pir* lysogen | [[2](#_ENREF_2)] |
| *Pseudomonas putida* |  |  |
| PaW85 | Wild type, isogenic to KT2440 | [[3](#_ENREF_3)] |
| *colR* | PaW85 *colR*::Km (Km^r^) | [[4](#_ENREF_4)] |
| *colS* | PaW85 *colS*::Km (Km^r^) | [[4](#_ENREF_4)] |
| RtacR | *colR* mutant containing genomic *lacI*^q^-*P*_tac_*-colR* expression cassette (Km^r^ Tel^r^) | [[4](#_ENREF_4)] |
| RtacR_D51A_ | *colR* mutant containing genomic *lacI*^q^-*P*_tac_*-colR_D51A_* expression cassette (Km^r^ Tel^r^) | [[4](#_ENREF_4)] |
| StacS | *colS* mutant containing genomic *lacI*^q^-*P*_tac_*-colS* expression cassette (Km^r^ Sm^r^) | This study |
| StacS_H35A_ | *colS* mutant containing genomic *lacI*^q^-*P*_tac_*-colS_H35A_* expression cassette (Km^r^ Sm^r^) | This study |
| StacS_E38Q_ | *colS* mutant containing genomic *lacI*^q^-*P*_tac_*-colS_E38Q_* expression cassette (Km^r^ Sm^r^) | This study |
| StacS_D57N_ | *colS* mutant containing genomic *lacI*^q^-*P*_tac_*-colS_D57N_* expression cassette (Km^r^ Sm^r^) | This study |
| StacS_H95A_ | *colS* mutant containing genomic *lacI*^q^-*P*_tac_*-colS_H95A_* expression cassette (Km^r^ Sm^r^) | This study |
| StacS_E96Q_ | *colS* mutant containing genomic *lacI*^q^-*P*_tac_*-colS_E96Q_* expression cassette (Km^r^ Sm^r^) | This study |
| StacS_H105A_ | *colS* mutant containing genomic *lacI*^q^-*P*_tac_*-colS_H105A_* expression cassette (Km^r^ Sm^r^) | This study |
| StacS_E126Q_ | *colS* mutant containing genomic *lacI*^q^-*P*_tac_*-colS_E126Q_* expression cassette (Km^r^ Sm^r^) | This study |
| StacS_E129Q_ | *colS* mutant containing genomic *lacI*^q^-*P*_tac_*-colS_E129Q_* expression cassette (Km^r^ Sm^r^) | This study |
| StacS_E126Q/E129Q_ | *colS* mutant containing genomic *lacI*^q^-*P*_tac_*-colS_E126Q/E129Q_* expression cassette (Km^r^ Sm^r^) | This study |
| Δ33-35 | PaW85 ΔPP0033-PP0035 | This study |
| 268 (*oprQ*) | PaW85 PP0268::Sm (Sm^r^) | This study |
| Δ737 (Δ*pagL*) | PaW85 ΔPP0737 | This study |
| 900 | PaW85 PP0900::Sm (Sm^r^) | This study |
| Δ903-905 | PaW85 ΔPP0903-PP0905 | This study |
| 1636 (*dgkA*) | PaW85 PP1636::Sm (Sm^r^) | This study |
| Δ2579 (Δ*cptA*) | PaW85 ΔPP2579 | This study |
| 5152 | PaW85 PP5152::Sm (Sm^r^) | This study |
| Δ33-35; 900 | ΔPP0033-PP0035 and PP0900::Sm (Sm^r^) | This study |
| Δ33-35; Δ903-905 | ΔPP0033-PP0035 and ΔPP0903-PP0905 | This study |
| Δ33-35; Δ2579 | ΔPP0033-PP0035 and ΔPP2579 | This study |
| Δ903-905; Δ2579 | ΔPP0903-PP0905 and ΔPP2579 | This study |
| Δ33-35; Δ2579; Δ903-905 | ΔPP0033-PP0035 and ΔPP2579 and ΔPP0903-PP0905 | This study |
| Δ33-35; Δ2579; Δ903-905; 900 | ΔPP0033-PP0035 and ΔPP2579 and ΔPP0903-PP0905 and PP0900::Sm (Sm^r^) | This study |
| Δ33-35; Δ2579; Δ903-905; 5152 | ΔPP0033-PP0035 and ΔPP2579 and ΔPP0903-PP0905 and PP5152::Sm (Sm^r^) | This study |
| *colR*; 268 | *colR* mutant with disrupted PP0268::Sm (Km^r^ Sm^r^) | This study |
| *colR*; Δ737 | *colR* mutant with deleted PP0737 | This study |
| Plasmids |  |  |
| p9TT_B_lacZ | Promoter-probe pPR9TT derivative containing full-length *lacZ* (Amp^r^ Cm^r^) | [[5](#_ENREF_5)] |
| p9TT_B_lacZ/35 | PP0035 promoter fused with *lacZ* in p9TT_B_lacZ (Amp^r^ Cm^r^) | [[6](#_ENREF_6)] |
| p9TT_B_lacZ/268 | PP0268 promoter fused with *lacZ* in p9TT_B_lacZ (Amp^r^ Cm^r^) | [[5](#_ENREF_5)] |
| p9TT_B_lacZ/900 | PP0900 promoter fused with *lacZ* in p9TT_B_lacZ (Amp^r^ Cm^r^) | [[6](#_ENREF_6)] |
| p9TT_B_lacZ/*colR* | *colR* promoter fused with *lacZ* in p9TT_B_lacZ (Amp^r^ Cm^r^) | [[6](#_ENREF_6)] |
| p9TT_B_lacZ/903 | PP0903 promoter fused with *lacZ* in p9TT_B_lacZ (Amp^r^ Cm^r^) | [[6](#_ENREF_6)] |
| p9TT_B_lacZ/737 | PP0737 promoter fused with *lacZ* in p9TT_B_lacZ (Amp^r^ Cm^r^) | [[6](#_ENREF_6)] |
| p9TT_B_lacZ/1636 | PP1636 promoter fused with *lacZ* in p9TT_B_lacZ (Amp^r^ Cm^r^) | [[6](#_ENREF_6)] |
| p9TT_B_lacZ/2579 | PP2579 promoter fused with *lacZ* in p9TT_B_lacZ (Amp^r^ Cm^r^) | This study |
| p9TT_B_lacZ/5152 | PP5152 promoter fused with *lacZ* in p9TT_B_lacZ (Amp^r^ Cm^r^) | This study |
| pBRlacItac | Expression vector containing *lacI^q^* repressor-controlled *P_tac_* promoter (Amp^r^) | [[7](#_ENREF_7)] |
| pBRlacItac/colS | pBRlacItac containing *colS* in a SalI-HincII fragment under the *P_tac_* promoter (Amp^r^) | This study |
| pUC18Not | pUC18 with NotI restriction sites in multicloning region (Amp^r^) | [[2](#_ENREF_2)] |
| pUCNot/lacItaccolS | pUCNot containing *lacI*^q^-*P*_tac_*-colS* expression cassette (Amp^r^) | This study |
| pUCNot/lacItaccolS_H35A_ | pUCNot containing *lacI*^q^-*P*_tac_*-colS_H35A_* expression cassette (Amp^r^) | This study |
| pUCNot/lacItaccolS_E38Q_ | pUCNot containing *lacI*^q^-*P*_tac_*-colS_E38Q_* expression cassette (Amp^r^) | This study |
| pUCNot/lacItaccolS_D57N_ | pUCNot containing *lacI*^q^-*P*_tac_*-colS_D57N_* expression cassette (Amp^r^) | This study |
| pUCNot/lacItaccolS_H95A_ | pUCNot containing *lacI*^q^-*P*_tac_*-colS_H95A_* expression cassette (Amp^r^) | This study |
| pUCNot/lacItaccolS_E96Q_ | pUCNot containing *lacI*^q^-*P*_tac_*-colS_E96Q_* expression cassette (Amp^r^) | This study |
| pUCNot/lacItaccolS_H105A_ | pUCNot containing *lacI*^q^-*P*_tac_*-colS_H105A_* expression cassette (Amp^r^) | This study |
| pUCNot/lacItaccolS_E126Q_ | pUCNot containing *lacI*^q^-*P*_tac_*-colS_E126Q_* expression cassette (Amp^r^) | This study |
| pUCNot/lacItaccolS_E129Q_ | pUCNot containing *lacI*^q^-*P*_tac_*-colS_E129Q_* expression cassette (Amp^r^) | This study |
| pUCNot/lacItaccolS_E126Q/E129Q_ | pUCNot containing *lacI*^q^-*P*_tac_*-colS_E126Q/E129Q_* expression cassette (Amp^r^) | This study |
| pBK-miniTn7-ΩSm | pUC19-based delivery plasmid for miniTn7-ΩSm (Amp^r^ Sm^r^) | [[8](#_ENREF_8)] |
| pminiTn7lacItac-colS | pBK-miniTn7-ΩSm containing *lacI*^q^-*P*_tac_-*colS* expression cassette (Amp^r^ Sm^r^) | This study |
| pminiTn7lacItac-colS_H35A_ | pBK-miniTn7-ΩSm containing *lacI*^q^-*P*_tac_-*colS_H35A_* expression cassette (Amp^r^ Sm^r^) | This study |
| pminiTn7lacItac-colS_E38Q_ | pBK-miniTn7-ΩSm containing *lacI*^q^-*P*_tac_-*colS_E38Q_* expression cassette (Amp^r^ Sm^r^) | This study |
| pminiTn7lacItac-colS_D57N_ | pBK-miniTn7-ΩSm containing *lacI*^q^-*P*_tac_-*colS_D57N_* expression cassette (Amp^r^ Sm^r^) | This study |
| pminiTn7lacItac-colS_H95A_ | pBK-miniTn7-ΩSm containing *lacI*^q^-*P*_tac_-*colS_H95A_* expression cassette (Amp^r^ Sm^r^) | This study |
| pminiTn7lacItac-colS_E96Q_ | pBK-miniTn7-ΩSm containing *lacI*^q^-*P*_tac_-*colS_E96Q_* expression cassette (Amp^r^ Sm^r^) | This study |
| pminiTn7lacItac-colS_H105A_ | pBK-miniTn7-ΩSm containing *lacI*^q^-*P*_tac_-*colS_H105A_* expression cassette (Amp^r^ Sm^r^) | This study |
| pminiTn7lacItac-colS_E126Q_ | pBK-miniTn7-ΩSm containing *lacI*^q^-*P*_tac_-*colS_E126Q_* expression cassette (Amp^r^ Sm^r^) | This study |
| pminiTn7lacItac-colS_E129Q_ | pBK-miniTn7-ΩSm containing *lacI*^q^-*P*_tac_-*colS_E129Q_* expression cassette (Amp^r^ Sm^r^) | This study |
| pminiTn7lacItac-colS_E126Q/E129Q_ | pBK-miniTn7-ΩSm containing *lacI*^q^-*P*_tac_-*colS_E126Q/E129Q_* expression cassette (Amp^r^ Sm^r^) | This study |
| pUXBF13 | Helper plasmid coding for the Tn7 transposition proteins (Amp^r^ *mob*^+^) | [[9](#_ENREF_9)] |
| pEMG | Plasmid for homologous recombination, *lacZα* with two flanking I-SceI sites (Km^r^) | [[10](#_ENREF_10)] |
| pEMG/Δ35-33 | pEMG containing the chimeric DNA fragment for deleting PP0035-PP0033 operon (Km^r^) | This study |
| pEMG/Δ737 | pEMG containing the chimeric DNA fragment for deleting *pagL*-encoding PP0737 (Km^r^) | This study |
| pEMG/Δ903-905 | pEMG containing the chimeric DNA fragment for deleting PP0903-PP00905 operon (Km^r^) | This study |
| pEMG/Δ2579 | pEMG containing the chimeric DNA fragment for deleting PP2579 (Km^r^) | This study |
| pSW (I-SceI) | Plasmid coding for I-SceI endonuclease for allelic exchange experiments (Bp^r^) | [[11](#_ENREF_11)] |
| pKS/268 | pBluescript KS containing PCR-amplified PP0268 locus (Amp^r^) | This study |
| pUTmini-Tn5Sm/Sp | Delivery plasmid for mini-Tn*5*Sm/Sp (Amp^r^ Sm^r^) | [[12](#_ENREF_12)] |
| pKS/268::Sm | Central 700 bp region of PP0268 in pKS/268 is replaced by Sm^r^ gene (Amp^r^ Sm^r^) | This study |
| pGP704L | Delivery plasmid for homologous recombination (Amp^r^) | [[13](#_ENREF_13)] |
| p704L/268::Sm | pGP704L with Acc65I-EcoRI fragment of 268::Sm from pKS/268::Sm (Amp^r^ Sm^r^) | This study |
| pRK2013 | Helper plasmid for conjugal transfer of pGP704L | [[14](#_ENREF_14)] |
| pKS/900 | pBluescript KS containing PCR-amplified PP0900 locus (Amp^r^) | This study |
| pKS/900::Sm | Central 87 bp region of PP0900 in pKS/900 is replaced by Sm^r^ gene (Amp^r^ Sm^r^) | This study |
| p704L/900::Sm | pGP704L with Acc65I-SacI fragment of 900::Sm from pKS/900::Sm (Amp^r^ Sm^r^) | This study |
| pKS/1636 | pBluescript KS containing PCR-amplified PP1636 locus (Amp^r^) | This study |
| pKS/1636::Sm | Central 143 bp region of PP1636 in pKS/1636 is replaced by Sm^r^ gene (Amp^r^ Sm^r^) | This study |
| p704L/1636::Sm | pGP704L with Acc65I-SacI fragment of 1636::Sm from pKS/1636::Sm (Amp^r^ Sm^r^) | This study |
| pKS/5152 | pBluescript KS containing PCR-amplified PP5152 locus (Amp^r^) | This study |
| pKS/5152::Sm | Central 377 bp region of PP5152 in pKS/5152 is replaced by Sm^r^ gene (Amp^r^ Sm^r^) | This study |
| p704L/5152::Sm | pGP704L with XbaI-PvuII fragment of 5152::Sm from pKS/5152::Sm (Amp^r^ Sm^r^) | This study |

References:

1. Hanahan D, Meselson M: **Plasmid screening at high colony density**. *Methods Enzymol* 1983, **100**:333-342.

2. Herrero M, de Lorenzo V, Timmis KN: **Transposon vectors containing non-antibiotic resistance selection markers for cloning and stable chromosomal insertion of foreign genes in gram-negative bacteria**. *J Bacteriol* 1990, **172**(11):6557-6567.

3. Bayley SA, Duggleby CJ, Worsey MJ, Williams PA, Hardy KG, Broda P: **Two modes of loss of the Tol function from *Pseudomonas putida* mt-2**. *Mol Gen Genet* 1977, **154**(2):203-204.

4. Hõrak R, Ilves H, Pruunsild P, Kuljus M, Kivisaar M: **The ColR-ColS two-component signal transduction system is involved in regulation of Tn*4652* transposition in *Pseudomonas putida* under starvation conditions**. *Mol Microbiol* 2004, **54**(3):795-807.

5. Kivistik PA, Putrinš M, Püvi K, Ilves H, Kivisaar M, Hõrak R: **The ColRS two-component system regulates membrane functions and protects *Pseudomonas putida* against phenol**. *J Bacteriol* 2006, **188**(23):8109-8117.

6. Kivistik PA, Kivi R, Kivisaar M, Hõrak R: **Identification of ColR binding consensus and prediction of regulon of ColRS two-component system**. *BMC Mol Biol* 2009, **10**:46.

7. Ojangu EL, Tover A, Teras R, Kivisaar M: **Effects of combination of different -10 hexamers and downstream sequences on stationary-phase-specific sigma factor sigma(S)-dependent transcription in *Pseudomonas putida***. *J Bacteriol* 2000, **182**(23):6707-6713.

8. Koch B, Jensen LE, Nybroe O: **A panel of Tn*7*-based vectors for insertion of the *gfp* marker gene or for delivery of cloned DNA into Gram-negative bacteria at a neutral chromosomal site**. *J Microbiol Methods* 2001, **45**(3):187-195.

9. Bao Y, Lies DP, Fu H, Roberts GP: **An improved Tn7-based system for the single-copy insertion of cloned genes into chromosomes of gram-negative bacteria**. *Gene* 1991, **109**(1):167-168.

10. Martinez-Garcia E, de Lorenzo V: **Engineering multiple genomic deletions in Gram-negative bacteria: analysis of the multi-resistant antibiotic profile of *Pseudomonas putida* KT2440**. *Environ Microbiol* 2011, **13**(10):2702-2716.

11. Wong SM, Mekalanos JJ: **Genetic footprinting with mariner-based transposition in *Pseudomonas aeruginosa***. *Proc Natl Acad Sci U S A* 2000, **97**(18):10191-10196.

12. de Lorenzo V, Herrero M, Jakubzik U, Timmis KN: **Mini-Tn*5* transposon derivatives for insertion mutagenesis, promoter probing, and chromosomal insertion of cloned DNA in gram-negative eubacteria**. *J Bacteriol* 1990, **172**(11):6568-6572.

13. Pavel H, Forsman M, Shingler V: **An aromatic effector specificity mutant of the transcriptional regulator DmpR overcomes the growth constraints of *Pseudomonas* sp. strain CF600 on para-substituted methylphenols**. *J Bacteriol* 1994, **176**(24):7550-7557.

14. Figurski DH, Helinski DR: **Replication of an origin-containing derivative of plasmid RK2 dependent on a plasmid function provided *in trans***. *Proc Natl Acad Sci U S A* 1979, **76**(4):1648-1652.
