# Supplementary material for: The ColRS signal transduction system responds to the excess of external zinc, iron, manganese, and cadmium
Source: BMC Microbiol. 2014 Jun 20;14:162. doi: 10.1186/1471-2180-14-162 (PMC4074579; doi:10.1186/1471-2180-14-162)
Supplement: Additional file 2: Table S2 — The oligonucleotides. [file 1471-2180-14-162-S2.docx]

Additional Table S2. The oligonucleotides

| Name | Sequence (5’-3’)^a^ | Use |
| --- | --- | --- |
| ColSSal | ATGGTCGACCGAGGGCCGCGATGGAGT | construction of pBRlacItac/colS |
| ColSHincII | CCGTTGACCTGATACAAGAAATTA | construction of pBRlacItac/colS |
| Smut1 | TTTGCCCTGATGAGCGCGCT | site-directed mutagenesis of *colS* |
| Smut2 | GCAACCAGCCAGCCAAGGAT | site-directed mutagenesis of *colS* |
| S_H35A | CGCTCTTCGACCAGGGCCACCGTGCCGAC | generating the H35A mutation |
| S_E38Q | GAAATCAGTCGCTCTTGGACCAGGTGCAC | generating the E38Q mutation |
| S_D57N | TCACTGACGCTGTTCATACGCAGCAG | generating the D57N mutation |
| S_H95A | GACCGCGGTTTCGCCGAGGTGTTCCG | generating the H95A mutation |
| S_E96Q | CGCGGTTTCCACCAGGTGTTCCGAGA | generating the E96Q mutation |
| S_H105A | CAGCTGTCCTACGCCGCCATGGTCGA | generating the H105A mutation |
| S_E126Q | CAGAGCGACTTCCAGGAGCGCGA | generating the E126Q mutation |
| S_E129Q | TTCGAGGAGCGCCAGCGCGTGCTTTTTGC | generating the E129Q mutation |
| S_E126Q_E129Q | CAGAGCGACTTCCAGGAGCGCCAGCGCGTGCTTTTTGC | generating the E129Q/E129Q double mutation |
| oprE3Bam | GCTGGATCCGCATAGCGTCG | construction of pKS/268 |
| oprE3Xho | ACTCTCGAGGCCGGGGCTGTTCGATAC | construction of pKS/268 |
| 900Kpn | CAGGGGTACCGCGGGGACA | construction of pKS/900 |
| colRATGXho | AGCTCGAGCATCGGTTTCTCCTGTGCG | construction of pKS/900 |
| PP1635lopp | CGAAAGCTTGCTGAACAAGCAGATC | construction of pKS/1636 |
| PP1636Kpn | AGAGGTACCAGACCTCGGCCATG | construction of pKS/1636 |
| 5152lopp | TAGCTGCAGACTCGACACAAGGATGG | construction of pKS/5152 |
| 5153lopp | CGCAAGCTTCGAGGACGTGACAGA | construction of pKS/5152 |
| 33EcoRI | TTAGAATTCCAGATTTGAAGATTTAACG | construction of Δ33-35 |
| 33lopp | AATGCGGAAACGCCTCGCAA | construction of Δ33-35 |
| 35pikk | TTGCGAGGCGTTTCCGCATAGCCTATGGAGGCAAAGGT | construction of Δ33-35 |
| 35SalI | ATTGTCGACAAACACGGGAGCACCAA | construction of Δ33-35 |
| murIBHI | CAGGGATCCAAGTTCGCAGCCTTGCT | construction of Δ737 |
| 737pikk | CCCAGACCTTTAATGCTGTGTCAGGCAAAGCGGTAGAAAG | construction of Δ737 |
| 738lopp | CACAGCATTAAAGGTCTGGG | construction of Δ737 |
| 738algERI | GGCGAATTCTCAACACCGGCACCAC | construction of Δ737 |
| 903SacI | TAGAGCTCACCCTGTATTTCGATGGC | construction of Δ903-905 |
| orf222alg | TTCAAGCTTGATGGGGCTACG | construction of Δ903-905 |
| 905jarel | GTAGCCCCATCAAGCTTGAATGCGTTAGCTGGAAAAGCC | construction of Δ903-905 |
| 905KpnI | TATGGTACCCAGGTCGGGCTGGTGA | construction of Δ903-905 |
| 2580SacI | CCGAGCTCATCATGACGCGCATCATT | construction of Δ2579 |
| cptApikk | AAGGTACGACGAGGCATACTACCGCAAGAATGGCTTGCG | construction of Δ2579 |
| 2578SalI | CAGGTCGACCGCCCACAGT | construction of Δ2579 |
| cptAees | GTATGCCTCGTCGTACCTT | construction of Δ2579 |
| PP2579alg | GCCAAGCTTACACCGGAGAAATACCAG | construction of p9TT_B_lacZ/2579 |
| PP2580alg | TAGAAGCTTGATCTTCTTACGACGG | construction of p9TT_B_lacZ/2579 |
| 5152alg | TCGAAGCTTTCTTTTACCCAC | construction of p9TT_B_lacZ/5152 |
| 5153lopp | CGCAAGCTTCGAGGACGTGACAGA | construction of p9TT_B_lacZ/5152 |

^a^ The sites of restriction enzymes used in cloning are underlined and mutated nucleotides are double underlined.
