# Supplementary material for: The ColRS signal transduction system responds to the excess of external zinc, iron, manganese, and cadmium
Source: BMC Microbiol. 2014 Jun 20;14:162. doi: 10.1186/1471-2180-14-162 (PMC4074579; doi:10.1186/1471-2180-14-162)
Supplement: Additional file 3: Table S3 — The oligonucleotide pairs used in two sequential PCRs for site-directed mutagenesis of colS. [file 1471-2180-14-162-S3.docx]

Additional Table S3. The oligonucleotide pairs used in two sequential PCRs for site-directed mutagenesis of *colS*

| Mutation | Primers for I PCR | Primers for II PCR |
| --- | --- | --- |
| H35A | S_H35A and Smut1 | I PCR product and Smut2 |
| E38Q | S_E38Q and Smut1 | I PCR product and Smut2 |
| D57N | S_D57N and Smut1 | I PCR product and Smut2 |
| H95A | S_H95A and Smut2 | I PCR product and Smut1 |
| E96Q | S_E96Q and Smut2 | I PCR product and Smut1 |
| H105A | S_H105A and Smut2 | I PCR product and Smut1 |
| E126Q | S_E126Q and Smut2 | I PCR product and Smut1 |
| E129Q | S_E129Q and Smut2 | I PCR product and Smut1 |
| E126Q/E129Q | S_E126Q_E129Q and Smut2 | I PCR product and Smut1 |
